# IVUS Guided Ostial Lesion Preparation With Sequential Cutting Balloon Inflations and Stent Placement—The ORCAS Technique

**DOI:** 10.1002/ccr3.71328

**Published:** 2025-10-18

**Authors:** Claudiu Ungureanu, Mihai Cocoi, Marouane Boukhris, Giuseppe Colletti, Adrien Jossart, Quentin Trefois, Gregor Leibundgut

**Affiliations:** ^1^ Department of Cardiology CHU Helora, Jolimont Hospital La Louvière Belgium; ^2^ Department of Cardiology “Niculae Stancioiu” Heart Institute Cluj‐Napoca Romania; ^3^ Department of Cardiology CHU Dupuytren Limoges France; ^4^ Department of Cardiology Clinique Saint Joseph, Vivalia Arlon Belgium; ^5^ Department of Cardiology University Hospital Basel Basel Switzerland

**Keywords:** aorto‐ostial coronary disease, calcified coronary disease, complex coronary percutaneous intervention, intravascular invasive coronary imagining, Rodin‐CUT

## Abstract

The ORCAS technique highlights the potential value of continuous intracoronary imaging during key procedural steps such as plaque preparation and ostial stent implantation. Real‐time IVUS guidance may support more precise assessment and execution, possibly improving procedural accuracy and consistency in complex aorto‐ostial lesions, particularly in heavily calcified coronary anatomy.

**Abbreviation**
ORCAS: Ostial Rodin Cut Angioplasty and Stenting

## 1 Introduction

Percutaneous coronary interventions (PCI) of calcified lesions, particularly those involving aorto‐ostial locations, remain technically challenging due to the high risk of suboptimal lesion preparation, stent underexpansion, and geographic miss. Intravascular ultrasound (IVUS) has proven instrumental in optimizing both lesion modification and stent implantation, yet its use is often limited to pre‐ and post‐procedural assessments rather than real‐time procedural guidance.

In this case, we describe the application of real‐time IVUS guidance throughout the entire intervention—from lesion preparation to stent deployment—in the treatment of a calcified ostial right coronary artery (RCA) lesion. Using the RODIN‐CUT technique [1], which involves multiple cutting balloon inflations to enhance plaque modification, the operator's decision‐making process was guided by sequential IVUS imaging, allowing for tailored procedural adjustments based on intraprocedural findings. Once optimal lesion preparation was confirmed, a critical step in the procedure was the precise positioning of the ostial stent. Real‐time IVUS played a pivotal role in this phase, guiding the operator to achieve full ostial coverage while minimizing aortic protrusion. This case highlights the unique benefits of continuous, real‐time IVUS guidance, enabling seamless procedural adaptation and enhanced precision during complex PCI.

## 2 Case History

**FIGURE 1:**
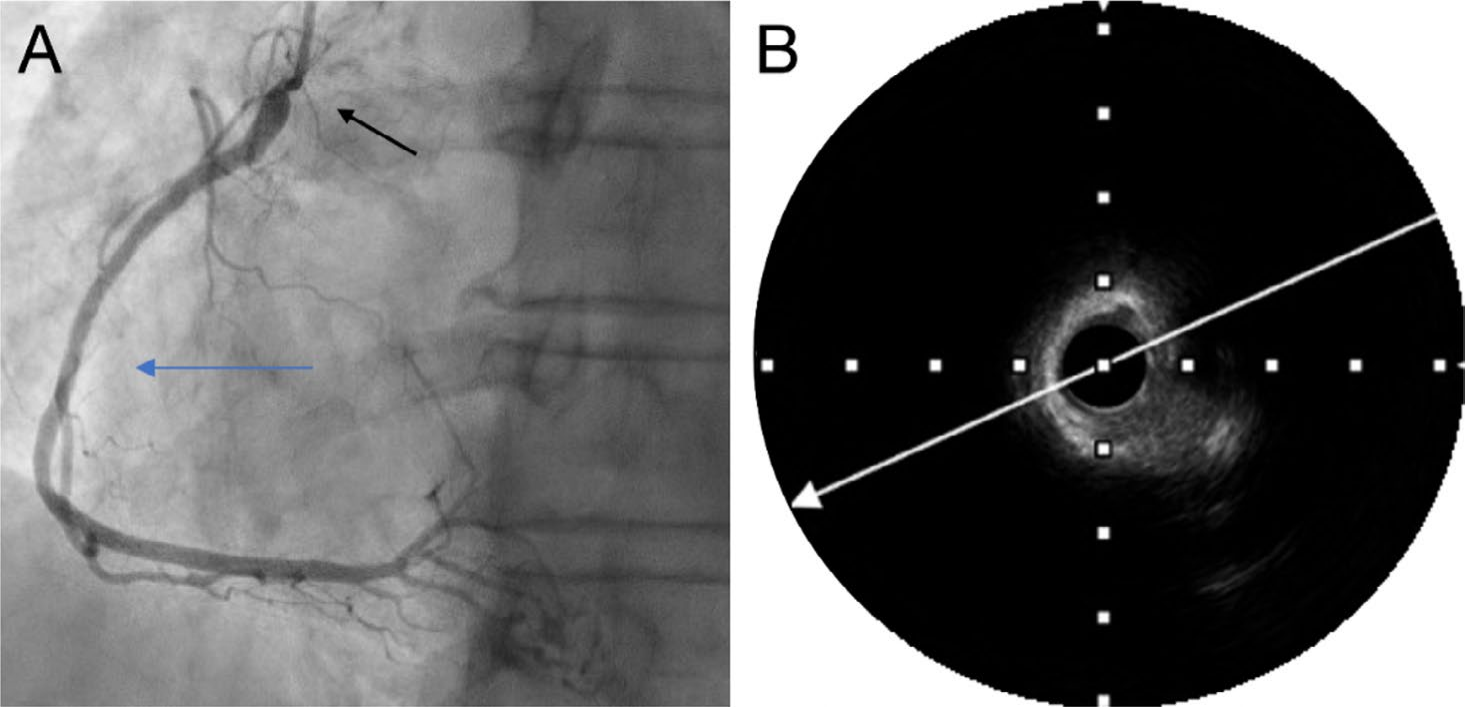
(A) Angiographic view—left cranial projection: Severe calcified and tight ostial lesion (dark arrow) of the right coronary artery (blue arrow). (B) Intravascular imaging (IVUS): Revealing a circumferential ring of dense calcium at the lesion site, consistent with a heavily calcified aorto‐ostial stenosis.

An 82‐year‐old female with a history of percutaneous coronary intervention (PCI) to the proximal right coronary artery (RCA) presented with recurrent chest pain. Coronary angiography revealed severe, concentric ostial RCA stenosis (Figure 1A). Given the complexity of the lesion—characterized by severe calcification and ostial involvement, both of which are associated with a high risk of target lesion revascularization (TLR)—meticulous lesion preparation was deemed essential.

## 3 Differential Diagnosis/Investigations and Treatment

PCI was performed via a right distal radial artery approach using the 7Fr RailTracking technique [2]. Initial IVUS evaluation confirmed a severely calcified and concentric ostial lesion that was not covered by the previously implanted stent, which showed only mild intimal proliferation (Figure 1B). Although FFR was not performed, the decision was based on symptoms and an IVUS‐derived MLA of 2.7 mm^2^, below accepted ischemic thresholds for the RCA.

**FIGURE 2:**
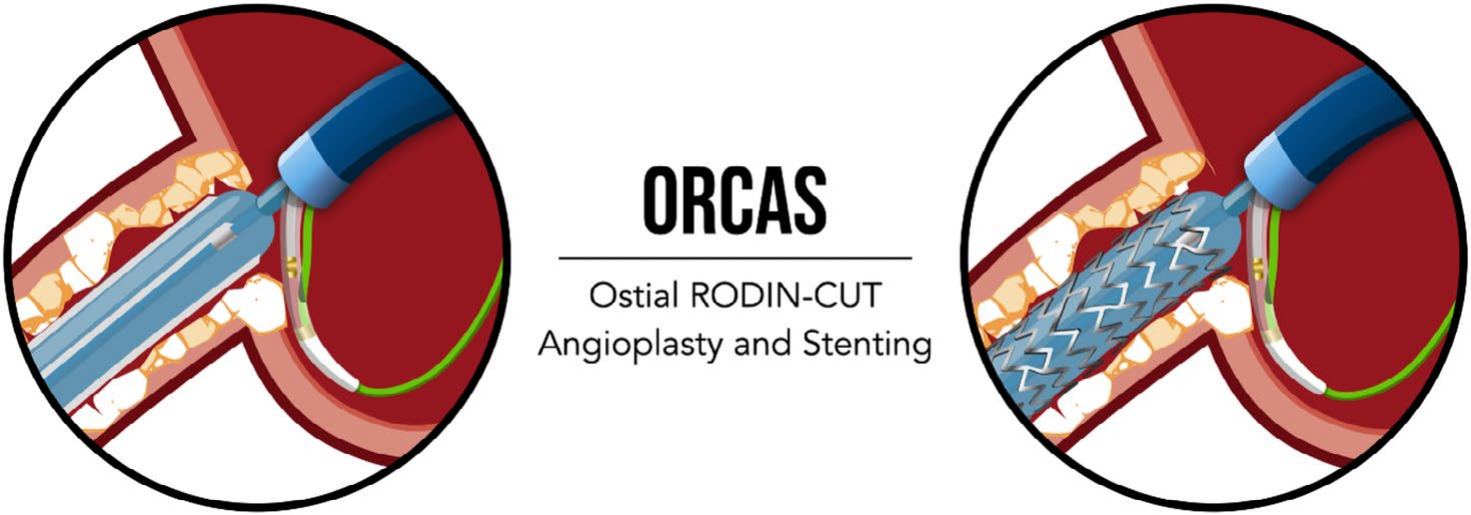
Left: Cutting balloon inflation using the RODIN‐CUT approach, demonstrating effective mechanical preparation of the calcified ostial lesion. Right: Real‐time IVUS‐guided stent positioning, with the IVUS catheter placed at the RCA ostium to ensure accurate alignment and avoid geographic miss.

**FIGURE 3:**
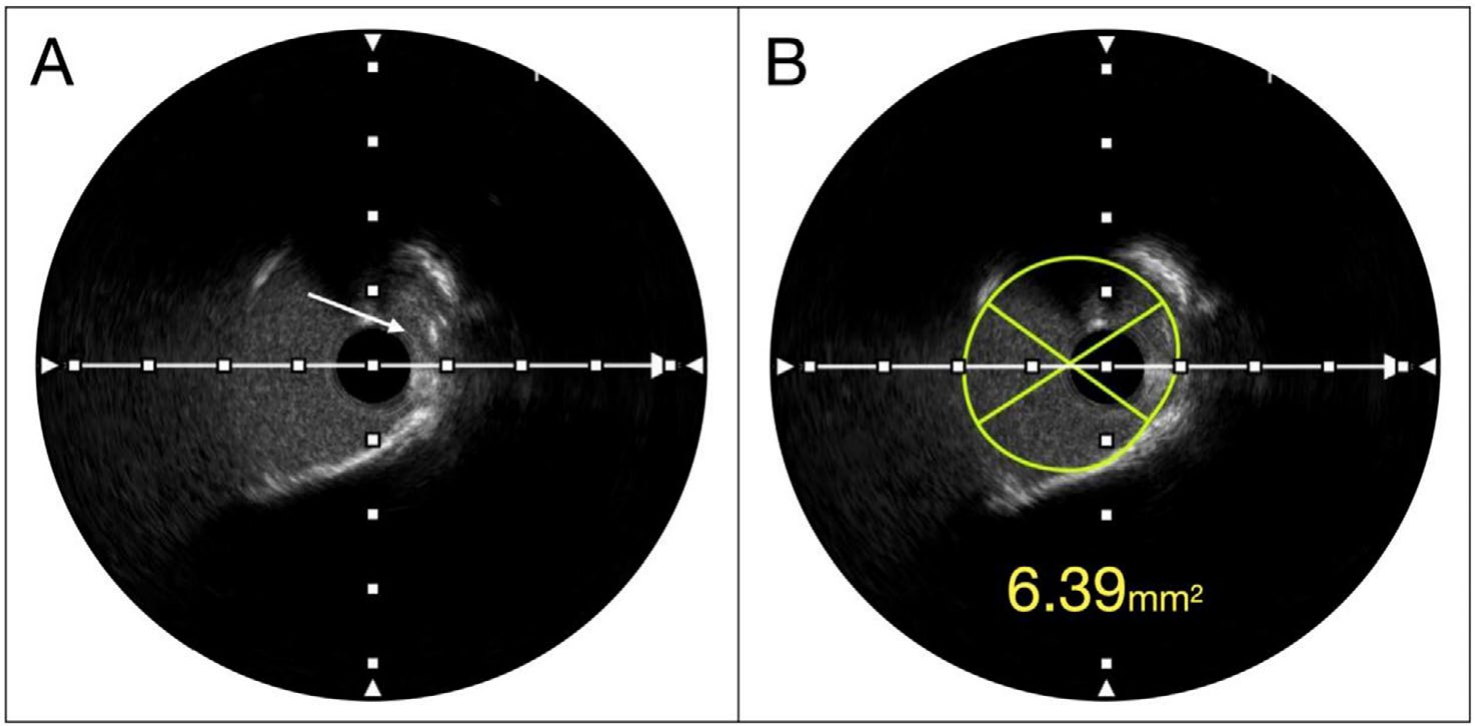
(A) IVUS image after multiple cutting balloon inflations showing visible calcium fractures and plaque disruption (white arrow), demonstrating a specific pattern entitled – “plaque decimation”. (B) Post‐preparation IVUS demonstrating significant increase in Minimal Lumen Area (MLA)confirming optimal lesion preparation using the RODIN‐CUT technique.

To optimize lesion preparation, the RODIN‐CUT technique [1] was employed using a 3.5/15 mm cutting balloon (CB). The lesion was sequentially prepared with high‐pressure inflations (18 atm), followed by real‐time IVUS pullbacks after each inflation to evaluate the extent of plaque modification (Figure 2). This iterative process allowed continuous assessment of plaque modification, ensuring the cutting balloon's efficacy in calcium modification and precisely identifying the optimal moment for stent implantation. Following five consecutive CB inflations, the minimal lumen area (MLA) improved significantly from 2.70 mm^2^ to 6.39 mm^2^ (Figure 3A), while the calcium arc of 270° was separated into smaller pieces of 100° and 90°, with clear evidence of plaque decimation indicating effective lesion preparation (Figure 3B).

**FIGURE 4:**
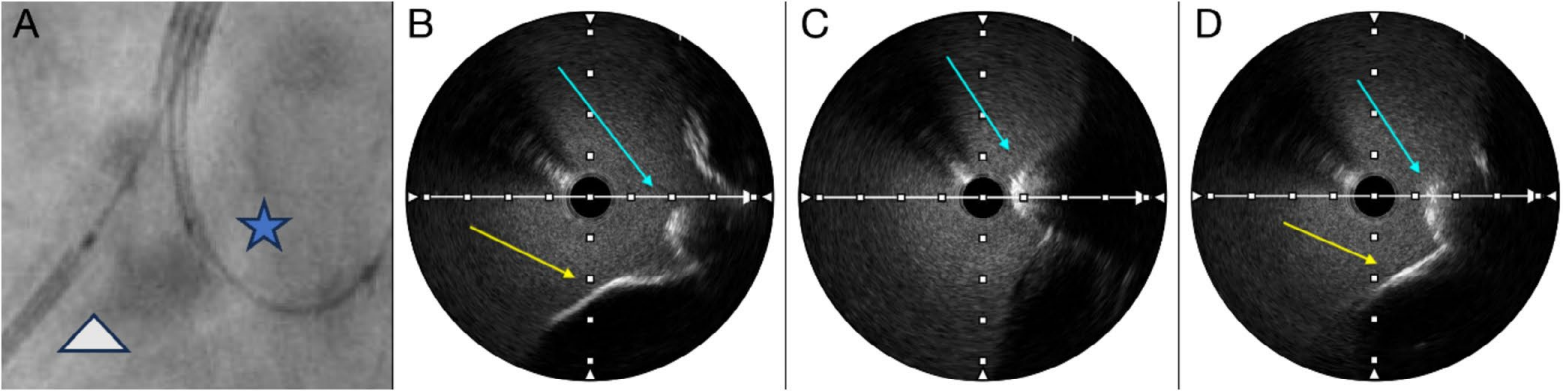
(A) Angiographic view of the RCA ostium with stent (gray triangle) positioned under guidance of an IVUS probe (blue star), advanced via a second guidewire into the right coronary cusp. (B) IVUS showing stent balloon tip too distal (light blue arrow), resulting in incomplete ostial coverage (yellow arrow) (geographic miss). (C) IVUS showing balloon tip excessively protruding into the aorta (light blue arrow), risking stent malposition. (D) IVUS confirming final optimal stent positioning (light blue arrow) with precise ostial coverage (yellow arrow) and minimal aortic protrusion.

Given the high takeoff of the RCA ostium, stent positioning using angiographic guidance alone was considered unreliable due to the high risk for geographic miss [3] (Figure 1A). To ensure precise placement, a second guidewire initially used for IVUS pullback was repositioned within the right coronary cusp, aligning the IVUS transducer directly in front of the RCA ostium (Figure 4A) [4]. IVUS imaging confirmed the proximal edge of the Megatron stent as a metallic structure with posterior shadowing and a visible “cap” (Figure 4B–D). By iteratively adjusting stent positioning under IVUS guidance, the operator ensured full ostial coverage without excessive aortic protrusion (Figure 4D).

**FIGURE 5:**
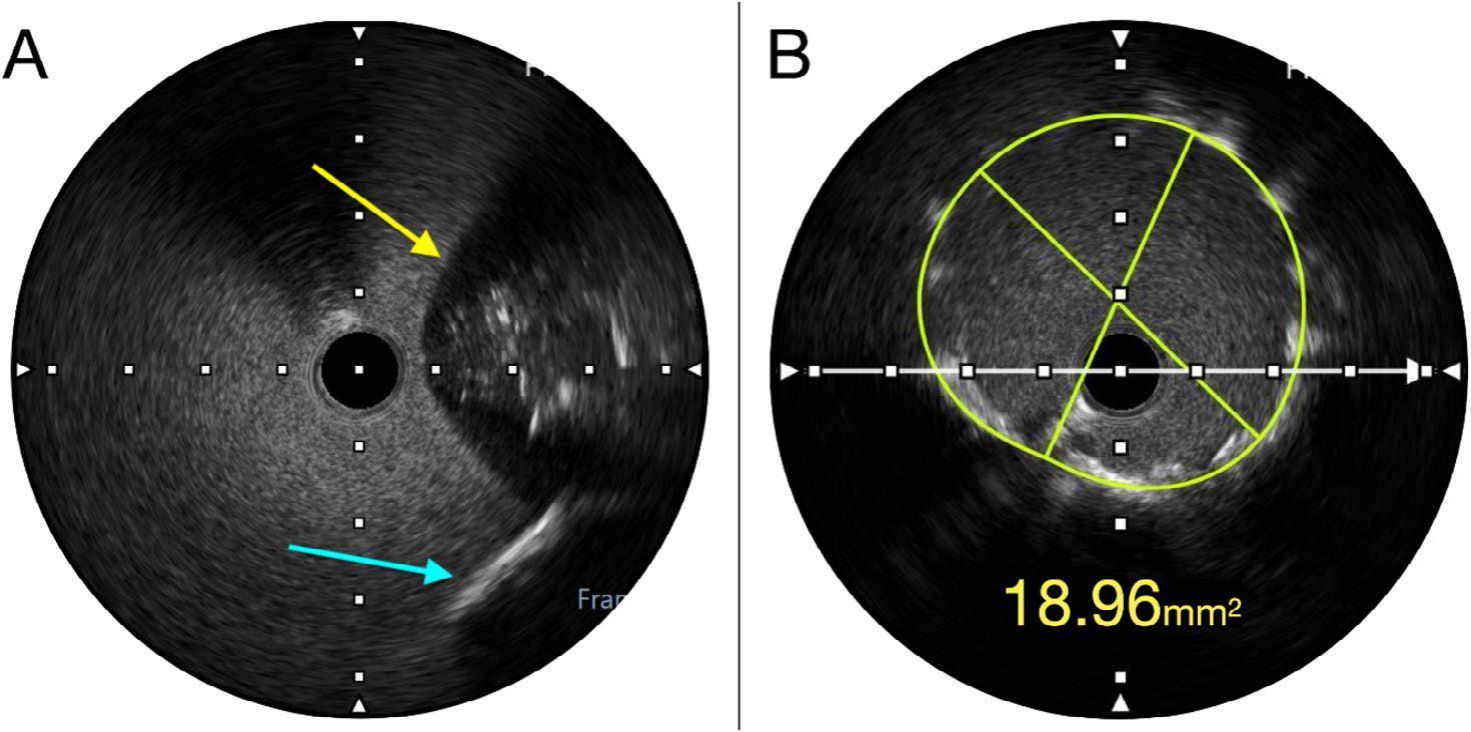
(A) IVUS image: Visualization of stent balloon inflation (yellow arrow) during deployment. Right coronary ostium is indicated by (light blue arrow). (B) IVUS image: Optimally expanded stent with a large Minimal Stent Area (MSA) following deployment.

**FIGURE 6:**
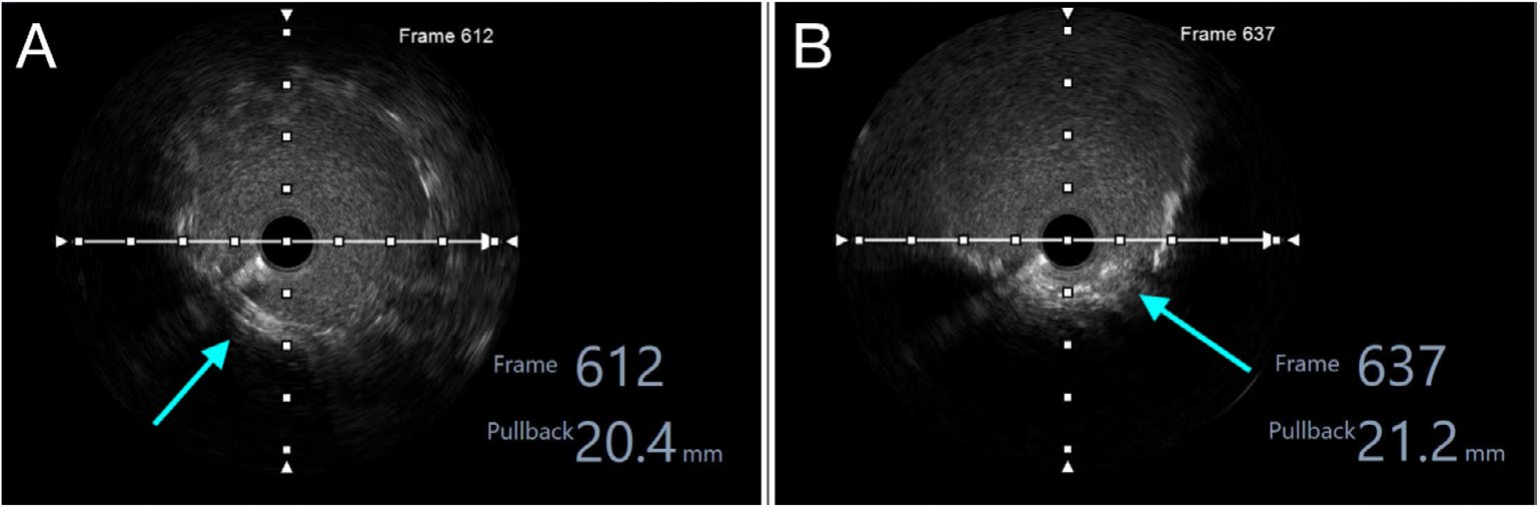
IVUS—Measurement of Stent Protrusion into the Aorta. (A) Proximal RCA segment just distal to the ostium, identified at frame 612 (20.4 mm from the start of pullback). (light blue arrow). (B) Last visible protruding stent struts in the ascending aorta at frame 637 (21.2 mm from the start), indicating a minimal stent protrusion of only 0.8 mm. (light blue arrow).

Upon stent deployment, IVUS imaging revealed a characteristic echolucent structure with bright internal reflections, confirming optimal stent balloon expansion and placement (Figure 5A). Final IVUS measurements demonstrated a large minimal stent lumen area (MSA) of 18.96 mm^2^ (Figure 5B), with minimal aortic protrusion (< 1 mm) (Figure 6A,B).

The clinical follow‐up at 6 months was uneventful, with the patient remaining asymptomatic and free of adverse cardiac events.

## 4 Discussion

This case underscores the pivotal role of continuous IVUS guidance during PCI, facilitating real‐time assessment of both plaque modification and stent positioning. The operator was able to systematically evaluate lesion response to each cutting balloon inflation, leading to optimal plaque preparation. Furthermore, direct intravascular visualization of anatomical landmarks enabled precise stent deployment at the RCA ostium, with intra‐procedural adjustments ensuring accurate alignment and minimal aortic protrusion.

While various techniques—such as the floating balloon [5] or the WALPO [6] techniques—offer effective solutions for ostial stent positioning, our case leveraged an alternative, IVUS‐guided approach. Since a second guidewire with the IVUS catheter was already in place, we simply repositioned it within the right coronary cusp, aligning the transducer directly with the RCA ostium for precise, real‐time stent placement, as previously described [7].

This IVUS‐guided strategy not only ensured perfect ostial alignment but also eliminated the guesswork associated with angiographic guidance alone, reinforcing the importance of IVUS as a continuous imaging tool for complex PCI. The procedural time was 48 min and radiation exposure remained low at 654 mGy, which is reassuring considering the complexity of the intervention.

Potential pitfalls of the technique should also be acknowledged. These include the requirement for a sufficiently large guide catheter to accommodate both the IVUS probe and the therapeutic device. Additionally, there is a risk of device friction, which could interfere with manipulation or exchange.

## 5 Conclusion and Results

The conventional use of IVUS in PCI improves clinical and procedural outcomes. Implementing real‐time IVUS guidance throughout all procedural steps further enhances precision, allowing real‐time assessment of lesion preparation and stent deployment. This dynamic approach has the potential to improve plaque modification, stent positioning, and ultimately procedural success in complex coronary interventions, particularly in calcified ostial lesions.

## Author Contributions

**Claudiu Ungureanu:** conceptualization, project administration, validation, writing – original draft, writing – review and editing. **Giuseppe Colletti:** writing – review and editing. **Mihai Cocoi:** conceptualization, methodology, writing – review and editing. **Marouane Boukhris:** conceptualization, validation, visualization, writing – review and editing. **Adrien Jossart:** visualization, writing – review and editing. **Quentin Trefois:** visualization, writing – review and editing. **Gregor Leibundgut:** conceptualization, supervision, writing – review and editing.

## Consent

Written informed consent was obtained from the patient for the publication of this case report and any accompanying images.

## Conflicts of Interest

The authors declare no conflicts of interest.

## Data Availability

The data that support the findings of this study are available on request from the corresponding author. The data are not publicly available due to privacy or ethical restrictions.
